# Appendectomy as a Potential Predisposing Factor for the Development of Recurrent and Fulminant Clostridium Difficile

**DOI:** 10.7759/cureus.10091

**Published:** 2020-08-28

**Authors:** Stacey E Heindl, Nicholas Tsouklidis

**Affiliations:** 1 Medicine, Avalon University School of Medicine, Willemstad, CUW; 2 Medicine, California Institute of Behavioral Neurosciences & Psychology, Fairfield, USA; 3 Health Care Administration, University of Cincinnati Health, Cincinnati, USA; 4 Medicine, Atlantic University School of Medicine, Gros Islet, LCA

**Keywords:** clostridium difficile, c.diff, c.difficile, infection, fulminant, gut-associated lymphoid tissue, appendix, appendectomy, recurrent, biofilm

## Abstract

This literature review assesses how the vermiform appendix has been considered a vestigial organ by many, but over the years, new research has allowed us to reconsider its potential purpose. Studies have indicated that the appendix plays an evident role in immune response and harbors a biofilm that may remain unaffected by gastrointestinal infections, such as infection with Clostridium difficile. Our research analyzes the prominent gut-associated lymphoid tissue (GALT) as a mechanism of defence in infection, as well as the robust biofilm that could aid in the reinoculation of beneficial bacteria within the colon. Furthermore, we wanted to determine if patients who have undergone a prior appendectomy, leading to decreased GALT and a lack of a bacterial reservoir, were predisposed to the development of Clostridium difficile, with particular emphasis in the recurrence and development of fulminant Clostridium difficile infections. Although research continues to be conflicting, there appears to be some connection between these variables, but prospective studies are needed in order to say for certain that there is a link.

## Introduction and background

The vermiform appendix has been a long-standing enigma regarding whether it is truly a vestigial organ, so much so that research has even hypothesized the various effects it can have on the severity and development of inflammatory bowel disease. In our research, we wanted to determine whether or not the function of the appendix could be providing a reservoir of bacteria for the colonic mucosa, thereby providing a stable microbiome that could aid in the protection against common gastrointestinal (GI) infections. Furthermore, we aimed to assess how the removal of the appendix could play a role in predisposing patients to Clostridium difficile infections (CDI), more specifically, the effect it could have on the initial onset of infection, recurrence and progression to fulminant CDI.

Vermiform appendix

The vermiform appendix is a small worm-like remnant of the cecum, thought to have no particular function; research has emerged over the years indicating this may not be the case. The appendix has a very similar histologic structure to the colon. Some studies indicate the appendix may provide more immune support than the colon itself [1-3].

**Figure 1:**
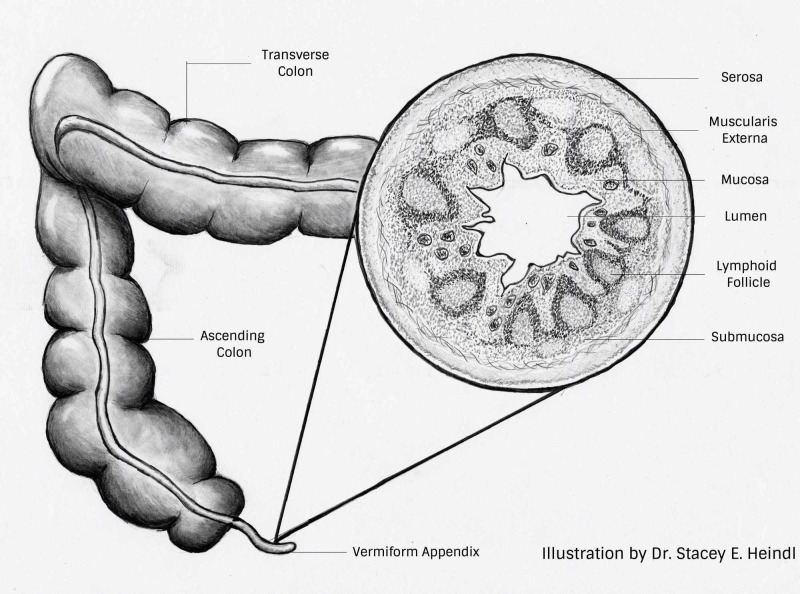
The anatomy and histology of the appendix

The histological structure of the appendix shows there is a mucosa, submucosa, muscularis externa and serosa (Figure 1). Within the layer of the submucosa, large lymphoid follicles are present, commonly referred to as gut-associated lymphoid tissue (GALT). These lymphoid follicles have an abundance of B lymphocytes and T lymphocytes, predominately found within the mantle region of the follicle. Within the germinal centre, follicular dendritic cells (FDC) and centrocytes can be found [1]. The histology is of importance, as we learn what is present within the follicles we can better understand how the appendix can provide an immune response. The presence of FDC allows for the presentation of antigens to lymphocytes in the form of antigen-antibody complexes, this promotes the lymphocytes to proliferate and differentiate into memory B cells and plasma cells, with the outcome being the production of specific antibodies against invading organisms [4]. When considering the histologic structure and function of the cells present, it is feasible to hypothesize how the appendix could play an essential role in fighting off unwarranted infections and building up antibodies to further protect against infection recurrence, such as in CDI.

Another critical area of interest is the ability of the appendix to produce a biofilm. Biofilm is composed of two parts, one being a thick mucin layer lining the colon. This layer is insoluble and helps provide a barrier to prevent the crossing of pathogens through the intestinal lining ultimately [1,5]. The second part of the biofilm is a thin mucin layer that overlays the thicker component [1]. The biofilm is a unique protective layer to the colon, as it not only provides a barrier but also helps shed bacteria from the colon after a diarrheal illness and provides reinoculation of beneficial bacteria to the colon [1,3,6,7]. This process allows for the ridding of pathogens from the body. Perhaps the most compelling aspect of this is that the appendix is the only area of biofilm not cleared after a diarrheal illness [1]. The ability to maintain this biofilm can provide us with further understanding of how the appendix could participate in the reinoculation of bacteria after CDI, potentially preventing the development of worsening illness to the point of becoming a fulminant infection. The aim of this literature review is to understand this concept better, as it could lead us in determining whether or not patients who have undergone an appendectomy are predisposed to recurrent CDI and the development of fulminant CDI due to the underlying lack of a biofilm reservoir and GALT.

Clostridium difficile

Clostridium difficile is a gram-positive, spore-forming bacterium that has been a threat primarily in hospitalized patients, accounting for close to 15% of all healthcare-associated infections in the United States [8]. Although this bacteria typically have a mild presentation, it can progress to become a more severe illness. Clostridium difficile can harbor two major virulence factors that play a significant role in the progression of this infection. Toxin A (TcdA) binds to the brush border of the cell wall and induces inflammation and fluid secretion, creating the typical symptoms we see in CDI, whereas Toxin B (TcdB) depolymerizes actin, furthermore disrupting cytoskeleton integrity, causing cell death and necrosis [9]. TcdB can also produce an exudate that creates a pseudomembrane leading to pseudomembranous colitis. Although most patients experience watery diarrhea with this illness, some patients can progress to fulminant CDI and present with systemic symptoms such as acute renal failure, respiratory distress, systemic inflammatory response syndrome (SIRS), hypotension, the elevation of leukocytes and lactate [9]. Unfortunately, after the progression to fulminant CDI, there is a risk of requiring a colectomy, this outcome ultimately changes one's prognosis to a mortality rate of around 67% [9]. Due to the potential of rapid progression of CDI, it is of the utmost importance to determine what population may be more susceptible to this and provide enhanced monitoring with possibly empiric therapy should symptoms begin to present.

## Review

Discussion

Clostridium difficile is a prominent pathogen typically found within the hospital, although community-acquired infections have also been reported. Resilience to harsh conditions such as chemicals, high temperatures, and antibiotics promotes its spread and its potential for recurrent infection [9]. Once the bacterium enters the GI tract, it can begin to thrive through the process of germination, followed by its adherence to the intestinal epithelium. Once this has taken place, toxins can begin to be released. Our goal was to evaluate each potential stage of this infection and how the appendix may play a role. We wanted to determine whether or not the appendix could affect the development of the primary infection, if it played a role in the recurrence of CDI, and if it could contribute to the progression to fulminant CDI.

Initial infection

Initial CDI can be due to a multitude of reasons such as poor hand hygiene, improper cleaning of hospital rooms, advanced age, proton pump inhibitor (PPI) use and antibiotic use, primarily with cephalosporins, clindamycin, and fluoroquinolones [10]. In our research, we wanted to determine if patients who had previously undergone an appendectomy were at an increased risk for the initial development of CDI. We believe the appendix could act as a bacterial reservoir, allowing for the continuous production of new biofilm to promote the shedding of harmful bacteria from the colon during a diarrheal illness. However, we do not believe there is a direct connection between the appendix being a protective factor in the onset of an initial infection. Merchant, et al. conducted a case-control study on this topic in 2012 with a total of 257 participants, 136 of which tested positive for CDI and 121 of which were negative for CDI. Merchant, et al. concluded that the rate of a prior appendectomy was lower in those with positive CDI, which would tell us that those with no appendix had lower rates of infection [11]. It also mentioned that 80% of the positive CDI cases were in those with an intact appendix [11]. The major limitation of this study is determining the real causality. Ward, et al. also analyzed the potential link between the appendix and CDI. Their study of 102 participants, 50 with positive CDI and 52 without CDI, showed that only 14% of patients with prior appendectomy developed CDI. Of the control group (negative CDI), 27% had a prior appendectomy, concluding no relationship between prior appendectomy and the development of CDI was found [12]. The high number of CDI cases in patients with an intact appendix found in Merchant, et al. study made us pose the question, could the appendix harbor Clostridium difficile during infection and promote the reinoculation of this bacterium into the colon, as opposed to aiding in the shedding of the bacteria? At this point, despite limited research on the topic and the lack of prospective studies, there is most likely no relationship between the initial development of CDI and prior appendectomy.

Recurrent Clostridium difficile infection

The relapse of CDI is becoming a more prominent problem within the healthcare field. Not only does recurrent infection place a burden on patients, but it also results in additional treatment costs. Patients typically experience a relapse of symptoms within 14 days after stopping antibiotic therapy for CDI [10]. As it stands, we know the appendix has an abundance of GALT and can provide a large production of biofilm, which is why we wanted to investigate further how this indeed plays a role in patients with recurrent CDI. Although the appendix may not have any direct relationship with the initial infection, its function could be a somewhat protective factor for recurrent infection. It is plausible to consider that after a CDI, the toxins produced will stimulate the B cells in the appendix to differentiate and form specific immunoglobulin G (IgG) and immunoglobulin M (IgM) to protect against recurrent infection [3]. Another way to view this would be in patients with prior appendectomy, there could be an impaired colonic immune response to CDI, leading to increased vulnerability to recurrent infection [13,14]. In addition, the production of biofilm from the appendix will promote the reinoculation of beneficial bacteria into the colon, further normalizing the microbiome to prevent recurrent infection and to enhance recovery. This mechanism could be viewed similarly to a colonic microbiota transplant used to restore the healthy flora in recurrent infections, supporting the idea that recurrence could be related to a depleted microbiome.

The current research regarding the appendix aiding in the prevention of recurrent CDI is conflicting. Im, et al. conducted a retrospective study in 2011 with 254 participants. Their study concluded that the presence of an appendix does have a significant and independent, inverse association with CDI recurrence [3]. Im, et al. goes a step further to discuss how previous studies have determined that there are even higher rates of recurrent CDI amongst patients who had undergone surgical procedures that included the removal of the appendix, such as an ileostomy and right hemicolectomy versus procedures where the appendix remained intact [3,15]. Na, et al. also reviewed this topic, suggesting the appendix may play a role in restoring colonization of the colon with beneficial bacteria, leading to fewer recurrences [16]. However, these studies need a prospective study for further validation.

In contrast, Fujii, et al. and Khanna, et al. concluded there is no difference in the course of illness, recurrence, or severity in patients with prior appendectomy versus an intact appendix [17,18]. These studies utilized 569 patients and 355 patients, respectively. However, as the majority of studies on this topic, they are both retrospective studies that are subject to confounding bias and difficulty in determining causation. It appears patients who have undergone an appendectomy may be predisposed to recurrent CDI, although the data is still conflicting. It would be difficult to determine the degree at which they are at risk and further analysis would be required.

Fulminant Clostridium difficile infection

The last pertinent area to discuss is the role the appendix could have as a protective factor in the development of fulminant CDI. As previously discussed, this is a more feared outcome of Clostridium difficile, as it can present with SIRS and progression to the potential need for surgical intervention. Similarly to recurrent CDI, we believe the appendix is protective in the development of fulminant infection, and those who have undergone an appendectomy could be predisposed to fulminant illness, with regards to the same mechanism seen in recurrent illness. Yong, et al. conducted a retrospective study with 507 patients, 388 with an intact appendix and 119 with a prior appendectomy. The results showed 5.2% of patients with an intact appendix developed a fulminant infection requiring a colectomy, whereas 10.9% with prior appendectomy required colectomy, concluding that appendectomy may be a risk factor for increased severity of CDI [19]. It should be noted that they did not determine a mechanism which would have led to these results. Clanton, et al. performed a retrospective cohort with 55 patients, stating there is statistical significance in the association of prior appendectomy and the development of fulminant CDI, resulting in colectomy [20]. The limitation of this study is the relatively small sample size. Due to the restricted number of studies on the subject, it would be premature to say that a prior appendectomy is a predisposing factor for the development of fulminant CDI (Table 1). These studies may be a good starting point with some evidentiary support to further assess the relationship being analyzed.

 

**Table 1:** Studies selected in our research CDI: Clostridium difficile infection

Study	Year	Study Type	Samples	Conclusion
Yong, et al. [19]	2015	Retrospective	507	5.2% with intact appendix developed fulminant infection. 10.9% with a prior appendectomy developed fulminant infection.
Fujii, et al. [17]	2013	Retrospective	569	No difference between course of diarrhea, recurrence or severity of CDI in patients with intact appendix versus prior appendectomy.
Khanna, et al. [18]	2013	Retrospective Cohort	355	Concluded there is no difference in treatment or outcomes between patients with prior appendectomy versus patients with intact appendix.
Clanton, et al. [20]	2013	Retrospective Cohort	55	Showed statistical significance between prior appendectomy and fulminated CDI resulting in colectomy.
Sanders, et al. [2]	2013	Review		Appendectomy most likely does not play a role in initial CDI but could play a role in preventing recurrent CDI.
Ward, et al. [12]	2013	Retrospective	102	No difference in the development of CDI and unable to determine if there is statistical difference in severity of disease.
Merchant, et al. [11]	2012	Retrospective Case-control	257	Believe there is no risk with prior appendectomy and that that appendix could harbor Clostridium difficile and promote inoculation into the colon.
Im, et al. [3]	2011	Retrospective	254	Presence of an appendix had a significant and independent, inverse association with CDI recurrence.
Na, et al. [16]	2011	Review		The appendix may accelerate restoration of colonization against CDI and lead to fewer recurrences. Patients with prior appendectomy may have impaired immune response to CDI.

Limitations

The major limitation of this paper is the minimal number of studies discussing the relationship between the appendix and Clostridium difficile infections. The majority of studies conducted were review articles and retrospective studies, which are not ideal as there is often confounding bias, and it can be difficult when trying to determine causality. Further research needs to be done prospectively to truly determine if patients who have undergone an appendectomy are at an increased risk of the development of recurrent and fulminant CDI.

## Conclusions

This review article aimed to analyze and discuss how a prior appendectomy may predispose patients not only to the development of CDI but also the recurrence and progression of the illness. It was shown that although there is likely no relationship between an appendectomy initiating the onset of CDI, there could be a correlation with the risk of recurrence and progression. Research has shown that the appendix participates in immunity and maintenance of the microbiome, which are relevant factors when analyzing what we know currently aids in the prevention and treatment of this illness. As stated, it is too early to determine if the correlation between these variables is strong enough to alter our current methods of monitoring and treatment, however, it should stress the importance of appendectomy only in indicated patients. Prospective studies must be done to clearly see if a relationship exists, and if it is of statistical and clinical significance, so we can better monitor and treat patients that could be at an overall higher risk in the development of this infection when compared to the general population. It would also be of interest to further investigate if a prior appendectomy makes patients more susceptible to particular strains of Clostridium difficile. 
